# Health impacts of a randomized biomass cookstove intervention in northern Ghana

**DOI:** 10.1186/s12889-021-12164-y

**Published:** 2021-12-04

**Authors:** Mona Abdo, Ernest Kanyomse, Rex Alirigia, Evan R. Coffey, Ricardo Piedrahita, David Diaz-Sanchez, Yolanda Hagar, Daniel J. Naumenko, Christine Wiedinmyer, Michael P. Hannigan, Abraham Rexford Oduro, Katherine L. Dickinson

**Affiliations:** 1 Colorado School of Public Health, Aurora, USA; 2 Navrongo Health Research Centre, Navrongo, Ghana; 3 Department of Mechanical Engineering, University of Colorado Boulder, Boulder, USA; 4 Berkeley Air Monitoring Group, Fort Collins, USA; 5 Environmental Protection Agency Human Studies Facility, Chapel Hill, USA; 6 Department of Applied Mathematics, University of Colorado Boulder, Boulder, USA; 7 Department of Anthropology, University of Colorado Boulder, Boulder, USA; 8 Institute of Behavioral Science, University of Colorado Boulder, Boulder, USA; 9 Cooperative Institute for Research in Environmental Sciences, University of Colorado Boulder, Boulder, USA

**Keywords:** Household air pollution, Cookstoves, Inflammation, Anthropometrics

## Abstract

**Background:**

Household air pollution (HAP) from cooking with solid fuels has adverse health effects. REACCTING (Research on Emissions, Air quality, Climate, and Cooking Technologies in Northern Ghana) was a randomized cookstove intervention study that aimed to determine the effects of two types of “improved” biomass cookstoves on health using self-reported health symptoms and biomarkers of systemic inflammation from dried blood spots for female adult cooks and children, and anthropometric growth measures for children only.

**Methods:**

Two hundred rural households were randomized into four different cookstove groups. Surveys and health measurements were conducted at four time points over a two-year period. Chi-square tests were conducted to determine differences in self-reported health outcomes. Linear mixed models were used to assess the effect of the stoves on inflammation biomarkers in adults and children, and to assess the z-score deviance for the anthropometric data for children.

**Results:**

We find some evidence that two biomarkers of oxidative stress and inflammation, serum amyloid A and C-reactive protein, decreased among adult primary cooks in the intervention groups relative to the control group. We do not find detectable impacts for any of the anthropometry variables or self-reported health.

**Conclusions:**

Overall, we conclude that the REACCTING intervention did not substantially improve the health outcomes examined here, likely due to continued use of traditional stoves, lack of evidence of particulate matter emissions reductions from “improved” stoves, and mixed results for HAP exposure reductions.

**Clinical trial registry:**

ClinicalTrials.gov (National Institutes of Health); Trial Registration Number: NCT04633135; Date of Registration: 11 November 2020 – Retrospectively registered.

URL: https://clinicaltrials.gov/ct2/show/NCT04633135?term=NCT04633135&draw=2&rank=1

**Supplementary Information:**

The online version contains supplementary material available at 10.1186/s12889-021-12164-y.

## Background

Worldwide, approximately 3 billion people cook using solid fuels (e.g., wood, coal), producing household air pollution (HAP) that contributed to approximately 2.3–3.8 million premature deaths per year. Specific illnesses linked to HAP exposure include pneumonia, acute lower respiratory disease, stroke, ischemic heart disease, lung cancer and chronic obstructive pulmonary disease (COPD) [1–5]. Cleaner cooking practices have the potential to reduce HAP exposure, improving people’s health and saving lives. While many so-called “improved” biomass stoves have shown promising results in laboratory tests, measuring the effectiveness of these stoves in real world settings is essential to assess whether or not these stoves are living up to their potential and should be more widely promoted.

**Fig. 1:**
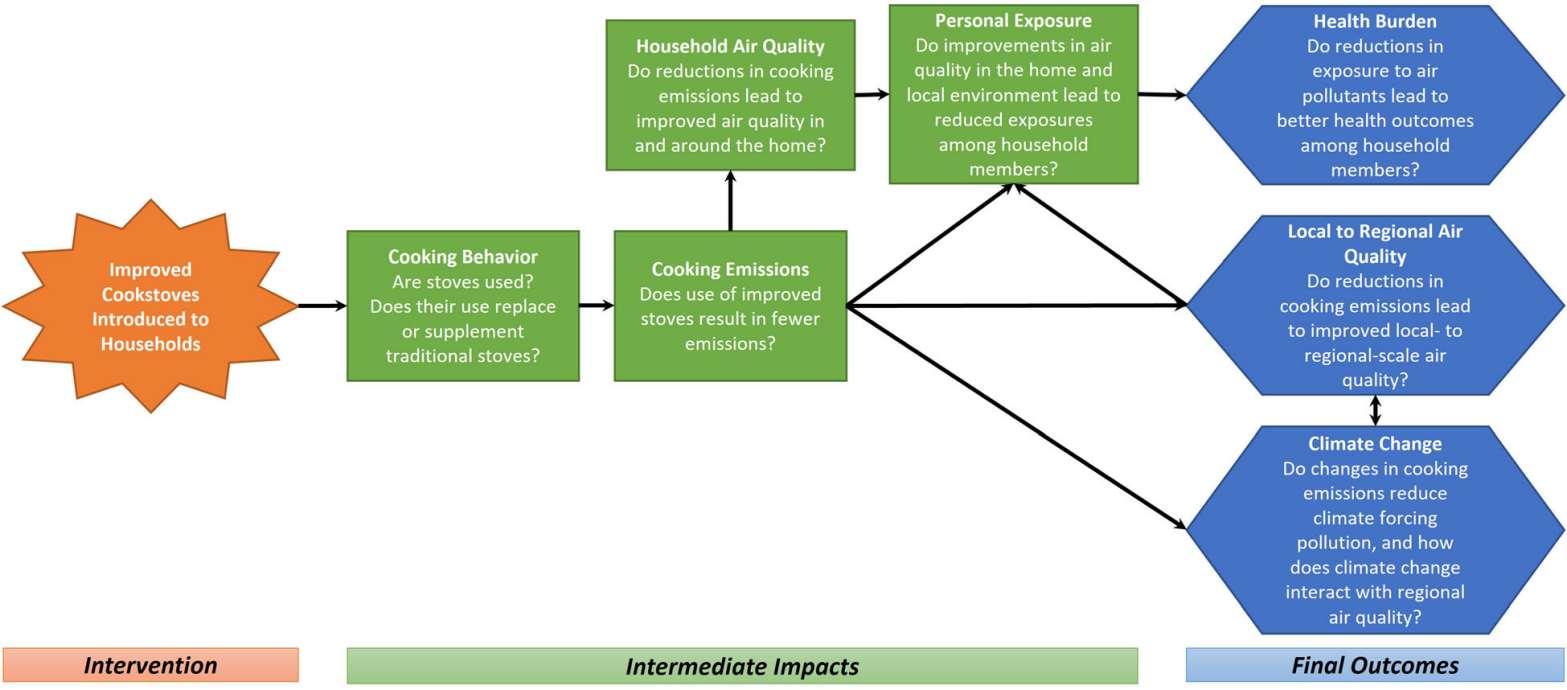
Logic model and measurements collected for the REACCTING study. Source: Dickinson et al. 2015 [6]

In this paper, we examine indicators of the health impacts of the REACCTING (Research on Emissions, Air Quality, Climate, and Cooking Technologies in Northern Ghana) randomized cookstove intervention study [6]. This study provided two different types of biomass stoves, which were both intended to improve cooking efficiency and reduce emissions, for free to study participants. Given limited evidence on the effectiveness of these stoves for changing cooking behaviors and reducing HAP exposure in a field setting, the primary focus of the REACCTING study was on these intermediate impacts on the causal pathways towards health and environmental outcomes (see Fig. 1).

In prior papers, we have reported effects of the intervention on cooking behavior [7, 8], stove emissions [9], fuel use [10], personal exposure [11, 12], and ambient air quality [11, 13]. Briefly, we found that households did *use* both types of improved stoves, but also continued to use their traditional wood and charcoal stoves (a practice known as stove stacking) [7, 8]. Use of the lower-tech rocket stove (Gyapa) was higher than use of the higher-tech gasifier stove (Philips), and use of the latter declined substantially in the second year of the study due in large part to battery failures [7]. Particulate matter *emissions* were not significantly reduced relative to a traditional three stone fire for either the Philips or the Gyapa stove, while the Philips stove did reduce carbon monoxide (CO) emissions by roughly 50% [9]. We also observed some reductions in personal *exposure* to particulate matter for all intervention groups, though results vary by pollutant; organic carbon exposure was reduced for all intervention groups, while elemental carbon was only reduced in groups receiving the Philips stove, relative to the control group [11].

Building on these results, this paper estimates effects of the REACCTING intervention on a set of health indicators. As noted above, studies have linked exposure to HAP with a wide variety of health outcomes. In particular, respiratory illnesses and pulmonary health outcomes have been a key focus of prior work [4, 5, 14]. A systematic review of studies that looked at the association between biomass fuel exposure and chronic obstructive pulmonary disease (COPD, clinically confirmed or self-reported) found that the overall odds were 2.4 [95% CI: 1.47,3.93] for women exposed to biomass fuel compared to other fuel exposure. In the same systematic review, children exposed to biomass fuel had 3.53 [95% CI: 1.94,6.443] times the odds of acute respiratory infection compared to children exposed to other types of fuel [15]. Another systematic review and meta-analysis also concluded that there was a positive association between solid fuel use and COPD (OR = 2.80, 95% CI = 1.85,4) [16]. Systematic reviews have also linked use of biomass fuels for cooking to lung cancer [17, 18]. Exposure to fine particulate matter air pollution, a key component of HAP from biomass fuel burning, has also been shown to contribute to cardiovascular disease and acute cardiac events [19].

A growing number of studies examine whether interventions aimed at reducing exposure to indoor pollutants can reduce the risk of adverse outcomes in both adults and children. The majority of the interventions studied have not been able to show a significant health impact. One notable exception is the RESPIRE study, a randomized controlled trial conducted in highland Guatemala among households with either a pregnant woman or young infants [20]. The intervention (woodstove with a chimney) led to a slight reduction in the incidence of pneumonia in children compared to the control (open woodfires) (RR = 0.82, 95%CI = 0.70,0.98) [20]. Meanwhile, the Cooking and Pneumonia Study (CAPS) in Malawi showed no significant differences between the intervention (gasifier woodstove) and control groups for pneumonia episodes in children (IRR = 1.01, 95% CI = 0.91,1.13) [21]. Similarly, another study in Orissa, India, concluded that the cookstove intervention did not have a significant impact on health outcomes [22]. In both of the latter cases, low rates of improved stove use contributed to small reductions in personal exposure to pollutants, limiting the potential for health effects. Stove uptake was higher among households with pregnant women in the Ghana Randomized Air Pollution and Health Study (GRAPHS) who received either a liquefied petroleum gas (LPG) stove or an improved biomass stove. Air pollution (CO and particulate matter) were reduced in the LPG arm relative to the control, but these reductions did not lead to significant improvements in birthweight [23].

In the REACCTING study, we did not have access to data on clinical cases of respiratory infections, pneumonia, COPD, birthweight, or other illnesses. To assess whether observed changes in cooking behavior and exposure were able to “move the needle” on health outcomes, we therefore relied on three primary sets of data measuring health outcomes associated with HAP exposure: self-reported health symptoms, biomarkers of inflammation collected from dried blood spots, and child growth measures from anthropometrics. Here we discuss each of these measures and its connection to HAP-related health outcomes.

### Self-reported health symptoms

In the absence of clinical health outcome data, studies in many contexts ask participants to report symptoms of health outcomes linked to the intervention under study. In the cookstove context, several studies have assessed impacts of improved stoves on self-reported symptoms associated with stove use and HAP exposure, including symptoms of respiratory illness (e.g., cough, wheeze, difficulty breathing) as well as eye irritation, headaches, and burns [24–27]. For example, Bensch et al. [25] reported that an improved cookstove intervention in Senegal reduced the incidence of respiratory symptoms and eye problems among women responsible for cooking, and Clark et al. [24] found that Honduran women cooking with traditional stoves were more likely to report respiratory symptoms than those using improved stoves.

The accuracy and reliability of self-reported health symptoms has been debated in this and other contexts. Some sources find a fairly high correspondence between measures of self-reported health status and objective health measures and argue that these measures can be an inexpensive and reasonably accurate way to measure health status [28–30]. Other works explore sources of measurement error in self-reported symptoms, finding that these measures can suffer from reporting biases [31, 32]. In light of this ongoing debate, strong conclusions about health impacts based on self-reported symptoms alone should be avoided. However, in the absence of clinical illness data, and when used alongside other objective health measures, self-reports may provide useful information about individuals’ perceived health burden and how this may vary across intervention groups [24].

### Biomarkers of inflammation

Another approach to measuring health outcomes when clinical data are not available, or where sample sizes may be too small to detect effects on clinical cases, is to examine sub-clinical indicators that are linked to health outcomes of interest. One of the suspected mediators on the pathway between exposure to particulate matter and both pulmonary and cardiovascular health outcomes is oxidative stress and pulmonary inflammation [19, 33–36]. Oxidative stress interacts with local inflammatory responses, promoting pro-inflammatory cytokine production and macrophage activation [36, 37]. Inflammatory biomarkers that have been linked with air pollution exposure include C-reactive protein (CRP) [38–41], interleukin-6 (IL-6) [41], interleukin-8 (IL-8) [42], and tumor necrosis factor alpha (TNF-alpha) [41]. These and other inflammatory biomarkers have also been linked with adverse respiratory health outcomes. For example, a meta-analysis concluded that patients with COPD had higher levels of IL-6 compared to healthy controls [43], and another study found that patients with COPD had higher levels of IL-1B compared to their healthy controls [44]. Other studies indicate that these biomarkers, particularly IL-6, TNF-alpha, and CRP, are also linked with processes that spill over from the respiratory system to mediate acute and chronic cardiovascular disease [19]. Thus, these biomarkers may provide useful sub-clinical measures of inflammation and oxidative stress responses linked to HAP exposure. A small number of other HAP and cooking studies have measured biomarkers as a health endpoint [24, 45].

### Child growth outcomes

Exposure to HAP has also been linked to child growth outcomes, including stunting in early childhood [46, 47]. Negative deviance in height-for-age and weight-for-age z-scores over time, or prolonged time below -2SD of the mean based on WHO Growth Standards, can be indicative of nutritional deficiencies [48], potentially as a result of exposure to HAP [49]. Inflammation has been shown to interact with hormonal regulators of growth and resultant linear growth in children, providing an indirect pathway by which HAP exposure might impact growth [50]. Thus, growth as a measure of health incorporates multiple pathways negatively affected by smoke exposure and can play an important role as a health status indicator.

The purpose of this study was to assess whether the REACCTING randomized cookstove intervention led to improved respiratory health indictors, including self-reported symptoms, biomarkers of inflammation, and child growth outcomes. Using each of these outcome measures, we test the hypothesis that participants randomly assigned to receive improved stoves will show improved respiratory health indicators relative to participants assigned to the control group, which continued to use traditional biomass stoves until the study’s conclusion.

## Methods

**Fig. 2:**
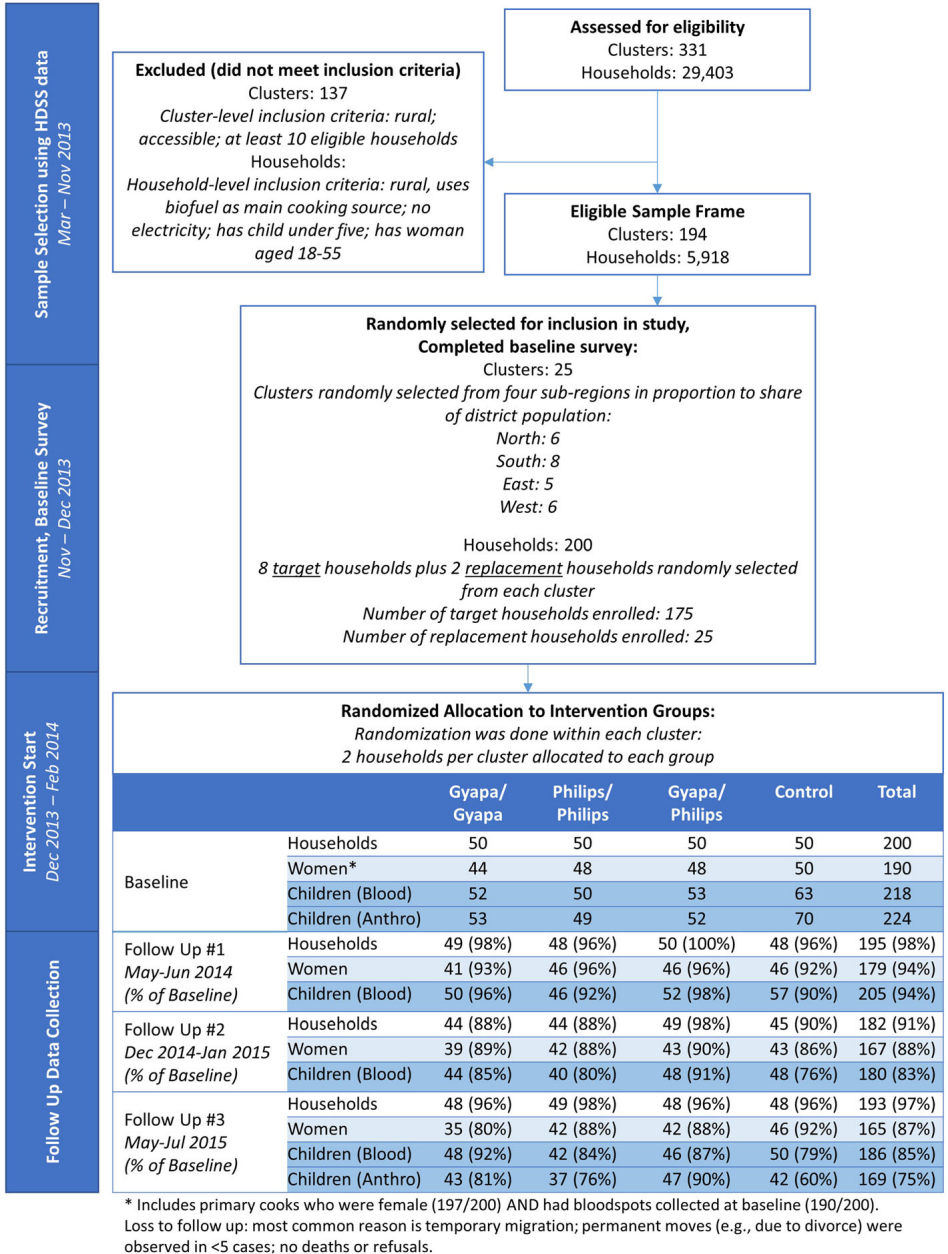
REACCTING Study Flow Diagram

The REACCTING study protocol is detailed in Dickinson et al. [6], and the study area is described by Oduro et al. [51]. In brief, the Kassena-Nankana Districts, located in the Upper East Region in Ghana, have a population of about 156,000 with an area of 1657 km^2^. The study was conducted through a partnership among researchers based in the United States and at the Navrongo Health Research Centre, an organization which routinely conducts a census of all households in the district (the Health and Demographic Surveillance Survey, HDSS). These data were used to carry out a cluster random sampling approach to select households for inclusion in the study (see Fig. 2). In this context, clusters consist of groups of up to 99 households defined for the purposes of HDSS data collection. The target population for the intervention included rural households that relied primarily on biomass for their cooking needs, and that included both adult women and young children (since these groups were widely thought to experience higher exposures to HAP). We thus used the HDSS data to apply a set of household-level eligibility criteria, which included: classified as “rural” according to the HDSS; reporting use of firewood, animal waste, or crop residue/sawdust as the household’s “main source of cooking fuel”; and having at least one child under five and one woman between the ages of 18 and 55 in the household. We then applied a set of cluster-level eligibility criteria: no more than 25% of households classified as urban according to the HDSS; accessible year-round (determined by field staff); and having at least 10 eligible households (after applying household-level criteria above). From the remaining set of clusters, we then randomly selected 25 from four geographic regions (North, South, East, West) in rough proportion to the distribution of population across these regions. Finally, we randomly selected eight target households and two replacements from each cluster. Target households were recruited for the study by the baseline interview team; if the household could not be located or declined to participate, a replacement was selected from the replacement list. The resulting sample consisted of 200 households.

The main traditional stove in the study area is a three stone fire and the majority of households also had a charcoal stove (“coal pot”). The REACCTING intervention involved two different types of improved biomass cookstoves, the Gyapa Woodstove and the Philips Smokeless Woodstove (HD4012-LS). Selection of these stoves for this intervention was based on formative research, described in more detail in the study protocol [6], which involved piloting stoves with households in the K-N Districts, assessment of stove testing results, and consultation with the international cookstove community. The Gyapa stove is a simple rocket stove that was made in Ghana specifically for this study. It represents a lower-tier, affordable stove with some potential for fuel efficiency improvements over a three stone fire. Meanwhile, at the time of the intervention’s launch, the Philips stove was widely regarded as the cleanest improved biomass stove model based on laboratory tests [52]. It had a forced draft design with a battery-powered fan (charged via a solar panel that was also provided to participants).

The study design involved randomizing the 200 participating households into one of four different cookstove groups, with randomization occurring *within* each cluster (i.e., stratified by cluster). Following the baseline survey, groups of households (eight households per cluster, as described above) were invited to community meetings where the two types of stoves were demonstrated and instructions on proper use were given. After the demonstration, randomization occurred by each participant (primary cook) drawing a slip of paper from a bucket with the intervention group assignment written on the paper. Within each cluster, two households were assigned to each of the four intervention arms. The first group received two Gyapa stoves, the second received two Philips stoves, the third received one of each type of stoves (one Gyapa and one Philips stove), and the fourth served as the control group for the duration of the study (and received their choice of two stoves at the study’s conclusion). Participants were not compensated beyond the stoves they received. The decision to provide two stoves to intervention households was based on our own prior work in this area and other studies that indicated a tendency for households to cook with multiple stoves and stove types [53], such that providing multiple stoves was thought to create a greater potential to displace traditional stoves.

### Outcome variables

Surveys and health measurements were conducted at four time points: baseline (Nov-Dec ‘13) and three follow up rounds, spaced about 6 months apart (May–June ‘14, Dec ‘14-Jan ‘15, and May-Jul ‘15). This region experiences a single rainy season lasting from approximately May to October; the timing of data collection thus corresponded roughly to the beginning of the dry season (Nov-Dec rounds) and the beginning of the rainy season (May–June rounds). Prior work has shown seasonal patterns in some health outcomes in this region (e.g., meningitis [54]), and cooking patterns (e.g., cooking indoors vs outdoors) also vary between the dry and rainy seasons. Our study design thus allows us to examine and control for possible seasonal effects in respiratory outcomes. Given the study’s focus on cooking behaviors and exposures, the main survey respondent was the household’s primary cook; measurements covered this individual and all children who were five years of age or younger at baseline, given that these individuals are widely thought to be more highly exposed to household air pollution in this setting. Survey instruments were developed by the study team; full instruments are publicly available online [55]. Surveys were conducted in the two local languages (Kassem and Nankam) by data collectors who were native speakers of these languages. Survey translation and back translation was completed by the Ghanaian research team, and inconsistencies were discussed and resolved with the broader team.

Self-reported health symptoms were collected at each survey. First, we included questions intended to measure current respiratory illness for the respondent (primary cook) and each child, asking whether these individuals experienced any of the following symptoms in the *past week*: runny nose, sore throat, difficulty breathing, wheezing or whistling in the chest, dry cough, cough with phlegm, or bloody cough. Respiratory symptom measures were drawn from other health surveys (e.g., the Indian National Family Health Survey [56]). Individuals reported to have any of these symptoms were coded as experiencing a respiratory illness. We also asked whether cooks and children currently had any burns from cooking, and whether respondents (primary cooks) experienced frequent headaches while cooking.

Given the potential for reporting bias in self-reported data, and because our relatively small sample was underpowered to detect changes in health outcomes like illness episodes, we collected sub-clinical indicators of individuals’ health status that may be linked to HAP exposure in the form of biomarkers of inflammation. These were measured through dried bloodspots collected from study participants (primary cooks and children under five) at each time point. Dried blood spots were collected, stored, and shipped following US Centers for Disease Control and Prevention guidelines described in multiple prior studies [57–59]. Once dried, each card was placed in a small low gas-permeable Ziploc bag with at least two 4280, Tri-sorb desiccant packets and a 3HIC125 humidity indicator card. The samples were stored at -80C once received until extraction. Previous validation studies have examined the stability of samples under these conditions and shown that decreases in samples for the tested analytes have been under 3% over a 1-year period.

For analysis, samples were shipped to the US Environmental Protection Agency laboratory in North Carolina. The team that conducted these analyses did not receive any information about study participants beyond a unique ID code; that is, this team was blinded to participants’ treatment status. Samples were analyzed using the Meso Scale Discovery (Meso Scale Diagnostics, LLC, Rockville, MD) multiplex assay as previously described [57]. Two kits were utilized, one for markers of vascular injury (C-reactive protein (CRP, ng/ml), Serum Amyloid A (SAA, ng/ml), soluble cell adhesion molecules (sCAMs), including sICAM (ng/ml), sVCAM (ng/ml)) and another for pro-inflammatory mediators (interleukins IL-1β (pg/ml), IL-6 (pg/ml), IL-8 (pg/ml), and tumor necrosis factor alpha (TNF-α, pg/ml)). A random sample of 1 in 20 samples were rerun in duplicate which allowed an estimation of both reliability (intra plate variability) and reproducibility (inter plate variability). In each case the coefficient of variation was between 5 and 10%. Multiple validation studies have been conducted comparing blood draws to dried blood spots. As an example, Clark et al. [24] report on a validation study of the modified dried blood spot CRP assay comparing CRP in plasma and dried blood spots from 40 volunteers. While the values were consistently higher in dried blood spots than plasma, there is a strong correlation between the two values [24].

For this analysis, these 8 biomarkers were used as the outcomes of interest and were included as continuous variables in the models. In addition, we created an aggregate immune response score to provide a single value representing immune activation at a given time point with the intent of reducing variation between inflammatory markers [60]. Z-scores were calculated for CRP (ng/ml), Serum Amyloid A (ng/ml), IL-1B (pg/ml), IL-6 (pg/ml), IL-8 (pg/ml), and TNF-a (pg/ml) for each dried blood spot and then summed. Any sample with fewer than 4 z-scores for aggregate calculation were excluded from any analysis involving the aggregate score. Such an additive aggregate index should increase in proportion to the extent of inflammation.

Anthropometric measures included weight (kg) and height (cm) taken at baseline and endline, and mid-upper arm circumference (cm) measured at all four time points. For children over 12 months, we measured *height* to the nearest 0.1 cm with a stadiometer, while *length* was measured with a tape measure for children under 12 months. (We refer to both of these measures as *height* through the rest of the paper.) Weight was measured to the nearest .1 kg with a digital scale. All three anthropometric measures were converted to z-scores (WAZ, HAZ, MUACZ) using the WHO Child Growth Standards [61, 62]. These z-scores indicate how many standard deviations an individual child is from a normal growth curve, based on age, with stunting and underweight defined as − 2 standard deviations below HAZ and WAZ, respectively.

### Statistical methods

Our analyses of health outcomes for adults (self-reported symptoms and biomarkers) are limited to female primary cooks that were included at baseline and had follow up data in at least one of the three follow up rounds. For children, self-reported health and biomarker analyses include children that were five and under at baseline, and had at least one follow round of data. Child growth outcomes are assessed for children that were five and under at baseline and had follow up data collected at endline (final round). The resulting sample sizes are presented in Table 1 and discussed in the next section.

**Table 1:** Baseline outcome and covariate levels for adult and children analysis samples, by intervention groups

	Variable	Full Sample	Gyapa/ Gyapa	Philips/ Philips	Gyapa/ Philips	Control
**ADULT WOMEN**	***Sample Size***	184	42	48	47	47
	Age (yrs) Mean (Range)	37.2 (18.4,82.6)	40.0 (19.6,82.6)	34.5 (18.7,56.6)	37.7 (18.6,68.6)	36.8 (18.4,73.6)
	Smoker in house (%)	33.1%	44.4%	35.7%	27.9%	26.2%
	Respiratory symptoms	43.5%	40.5%	43.8%	48.9%	40.4%
	Headache	67.8%	65.9%	72.9%	78.7%	53.2%
	Eye irritation	31.0%	31.0%	20.8%	40.4%	31.9%
	Burns	21.2%	14.3%	20.8%	25.5%	23.4%
	CRP (ng/ml)	42.0 (64.6)	24.8 (31.1)	50.9 (68.9)	43.3 (63.5)	47.1 (80.4)
	Serum Amyloid A (ng/ml)	146.7 (720.0)	70.2 (126.5)	282.6 (1352.5)	114.6 (235.5)	108.2 (310.3)
	Sicam (ng/ml)	12.2 (6.5)	11.1 (4.1)	12.9 (6.4)	13.0 (7.7)	11.9 (7.0)
	sVCAM (ng/ml)	19.5 (8.0)	19.9 (9.5)	19.4 (7.3)	19.5 (8.0)	19.2 (7.6)
	IL-1β (pg/ml)	0.7 (0.9)	0.90 (1.34)	0.72 (0.75)	0.59 (0.48)	0.71 (0.89)
	IL-6 (pg/ml)	0.09 (0.05)	0.08 (0.03)	0.11 (0.08)	0.09 (0.03)	0.08 (0.04)
	IL-8 (pg/ml)	7.7 (4.0)	7.5 (2.7)	8.02 (5.0)	7.7 (4.2)	7.4 (3.7)
	TNF-a (pg/ml)	0.2 (0.1)	0.2 (0.1)	0.2 (0.1)	0.2 (0.1)	0.2 (0.1)
**CHILDREN – SELF-REPORTED HEALTH & BIOMARKERS**	***Sample Size***	211	50	49	53	59
	Age (yrs) Mean (Range)	3.1 (0.8,5.2)	3.0 (1.1,5.2)	3.1 (1.0,5.0)	3.3 (1.2,5.1)	3.1 (0.8,5.1)
	Female (%)	49.3%	52.0%	53.1%	41.5%	50.9%
	Respiratory symptoms	50.2%	48.0%	51.0%	50.9%	50.9%
	CRP (ng/ml)	135.9 (294.9)	55.7 (93.3)	172.1 (308.9)	87.6 (179.0)	227.2 (428.8)
	Serum Amyloid A (ng/ml)	406.6 (1406.6)	98.1 (152.8)	354.5 (850.8)	165.0 (295.1)	928.2 (2460.1)
	sICAM (ng/ml)	19.4 (7.3)	18.4 (6.1)	19.6 (9.5)	19.9 (7.1)	19.7 (6.2)
	sVCAM (ng/ml)	28.0 (11.3)	26.9 (10.6)	25.8 (8.8)	27.5 (12.6)	31.3 (12.2)
	IL-1B (pg/ml)	0.34 (0.24)	0.31 (0.21)	0.34 (0.24)	0.34 (0.22)	0.35 (0.29)
	IL-6 (pg/ml)	0.14 (0.18)	0.11 (0.05)	0.14 (0.18)	0.14 (0.20)	0.17 (0.23)
	IL-8 (pg/ml)	10.8 (10.1)	9.8 (6.3)	9.8 (9.5)	9.9 (6.7)	13.4 (14.7)
	TNF-a (pg/ml)	0.43 (0.32)	0.42 (0.16)	0.41 (0.21)	0.46 (0.55)	0.43 (0.21)
**CHILDREN - ANTHRO**	***Sample Size***	184	49	40	43	52
	Age in yrs. Mean (Range)	3.1 (0.8, 5.2)	3.0 (1.1, 5.2)	3.0 (1.0, 5.0)	3.2 (1.3, 5.1)	3.2 (0.8, 5.1)
	Female (%)	49.5%	54.2%	53.7%	39.1%	51.0%
	HAZ	−1.46 (1.53)	−1.26 (1.68)	−1.58 (1.16)	−1.37 (0.89)	−1.62 (1.98)
	WAZ	−0.98 (1.34)	−1.00 (1.23)	−0.82 (1.14)	−0.83 (0.97)	− 1.18 (1.76)
	MUACZ	−0.54 (0.97)	−0.48 (1.01)	− 0.56 (1.00)	− 0.49 (0.87)	−0.64 (1.01)

ADULT WOMEN (top panel) sample includes all adult female primary cooks with blood spot measures at baseline and for at least one follow up round. CHILDREN – BLOOD SPOT (middle panel) sample includes children who were five and under at baseline, and had blood spot measures at baseline and at least one follow up round. CHILDREN – ANTHRO (bottom panel) sample includes children five and under at baseline, who had anthropometric measures at baseline and endline

Chi-square tests were conducted to determine differences in health symptoms (any respiratory, headaches, and burns) between stove groups in each survey round.

Mean (SD) and frequencies were used to describe the age and health symptoms of the adults and children in this analysis at baseline. For the biomarkers, we used linear mixed models to evaluate the association between stove group and levels of inflammatory biomarkers across different time points (Eq. 1).

1 $$ \ln \left({Y}_{ij}\right)\sim {\beta}_0+{\beta}_1 stovegr\mathrm{o}{up}_i+{\beta}_2{age}_{ij}+{\beta}_3{BMI}_i+{\beta}_4{sex}_i+{\beta}_5{time}_j+{\beta}_6{season}_j+{\beta}_7{smoke}_i+{\beta}_8\left(\ln \left({Y}_{i0}\right)\right)+{\upalpha}_i+{\varepsilon}_{ij} $$

*Y*_*ij*_ represents the biomarker level during for individual *i* in round *j*. A separate model was fit for each biomarker and for the aggregate scores. The models were fit separately for children and adults for a total of 18 models. The primary exposure variable included in the models was *stovegroup* (Gyapa/Gyapa, Philips/Philips, Gyapa/Philips, or Control (reference)). Other covariates included in the model included *age*, baseline *BMI*, *time* since baseline, and *season* (wet/dry). We also included an indicator for whether or not anyone in the household was reported to *smoke* tobacco. (While none of the respondents reported smoking, about one third said that someone else in the household smoked – see Table 1.) Since the purpose of these models is to assess whether people in different stove groups saw differential *changes* in their biomarker levels as a result of the intervention, baseline (log) levels of the biomarkers are included in these models (ln(*Y*_*i*0_)). Sex was only included in the children’s model since males were excluded from the adult analysis. Models including the pregnancy status of the mothers were also run, but due to the low number of cases and prevalence of missing values, this variable was not included in the models presented in the paper. An individual-level random intercept (α_*i*_) is included in these models to account for correlation within subjects. (For children, models were also run including household-level random effects to account for possible correlation within households; results were nearly identical to models without this effect, likely due to the fact that controlling for baseline values accounts for correlation in biomarkers within households at baseline.) Bonferroni corrections were conducted to account for multiple comparisons in the inflammatory marker models.

For child growth outcomes, we ran generalized linear models with each endline anthropometric measure as an outcome, correcting for baseline anthropometry, to determine if differential smoke exposure based on stove group impacted growth, controlling for sex and age of individual (Eq. 2). In this model, *Y*_1*i*_ represents the endline WAZ, HAZ, or MUACZ for individual *i*, while *Y*_0*i*_ represents this same measurement at baseline. Other variables are defined as in Eq. 1.

2 $$ {Y}_{1i}\sim {\beta}_0+{\beta}_1{stovegroup}_i+{\beta}_2{age}_i+{\beta}_3{sex}_i+{\beta}_4{Y}_{0i}+{\varepsilon}_{ij} $$

Finally, multinomial logistic regression models were fit to the final time point to assess if the stove group intervention had any impact on whether an individual was stunting or underweight, including sex, age, and whether or not the individual was breastfed at the survey timepoint as predictors (Eq. 3). Here, *Y*_1*i*_ is a binary indicator for whether individual i is stunting (<2SD median HAZ) or underweight (<2SD median WAZ) at endline. Breastfeeding was included as a categorical variable defined as children who were not breastfed at all during the course of the study, those that were breastfed for the entire study, and those that were breastfed only at baseline or endline.

3 $$ {Y}_{1i}\sim {\beta}_0+{\beta}_1{stovegroup}_i+{\beta}_2{age}_i+{\beta}_3{sex}_i+{\beta}_4{Y}_{0i}+{\beta}_5{breastfed}_i+{\varepsilon}_{ij} $$

Statistical analyses were performed using SAS (version 9.4), R (version 3.5.1), and JMP (version 14.0.0).

### Ethical considerations

The study protocol was reviewed and approved by Institutional Review Boards at the University of Colorado – Boulder, the National Center for Atmospheric Research, and the Navrongo Health Research Center.

## Results

### Sample characteristics

Table 1 reports characteristics of the samples used in our health outcome analyses. Of the 200 households included in the study at baseline, 197 had female primary cooks (adult women) (Fig. 2). Of these 197 women, 190 had biomarker data at baseline, and 184 of these had at least one round of follow up data collection; we assess self-reported health and biomarker outcomes for this sample of 184 adult women, and their characteristics are presented in the top panel of Table 1.

The second two panels of Table 1 show characteristics of children included in the biomarker and anthropometric samples, respectively. Across the 200 study households, a total of 218 children five and under had blood spots collected and 224 had anthropometric measurements at baseline (Fig. 2). For biomarkers and self-reported health symptoms, the analysis sample includes children five and under at baseline with at least one follow-up measurement, a total of 211 children. For anthropometrics, the sample includes children five and under at baseline with both baseline and endline (Round 4) measurements, a total of 184 children.

**Fig. 3:**
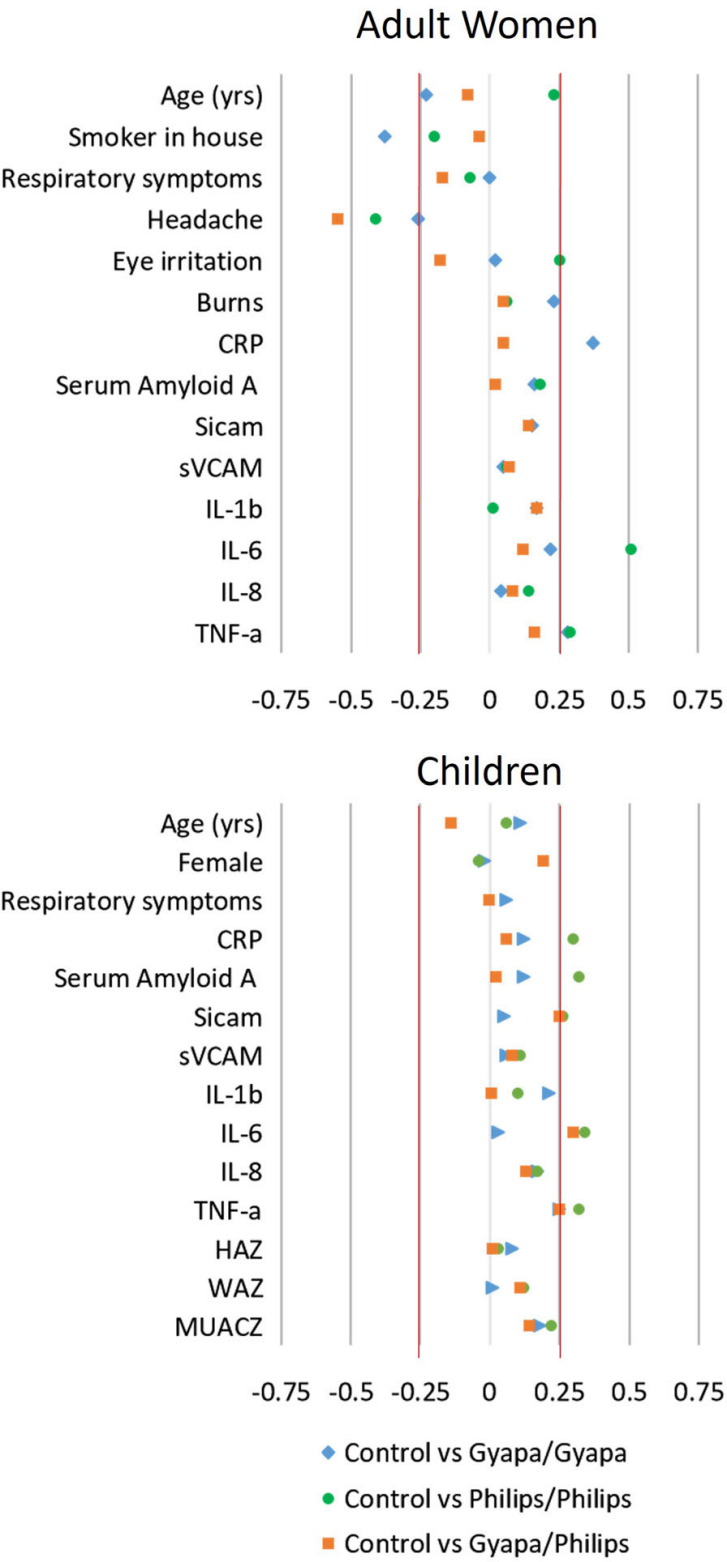
Standardized mean differences for covariates and outcome variables at baseline between intervention groups and control group. Values between − 0.25 and 0.25 (red lines) indicate well-balanced groups, while values outside this range indicate some degree of imbalance

To assess balance across treatment groups at baseline, we calculated standardized mean differences for each variable measured at baseline. This statistic is used in lieu of *p*-value comparisons, which are redundant when groups have been intentionally randomized. Standardized mean differences between − 0.25 and 0.25 indicate well-balanced groups, while larger values suggest some degree of imbalance [63]. Results are plotted in Fig. 3.

Despite randomization, we do observe some differences across intervention groups at baseline in a few key variables. For example, the proportion of cooks reporting headaches while cooking is somewhat lower in the control group (53%) compared to the Gyapa/Gyapa (66%), Philips/Philips (73%), and Gyapa/Philips (79%) groups. Among children, four of the inflammatory biomarkers (CRP, SAA, IL-6, and TNF-α) are somewhat imbalanced across treatment. Because randomization occurred in a transparent process, and because laboratory staff in charge of storing and analyzing the samples were blinded to treatment status, we are confident that these differences are due to chance. Nonetheless, it is important to account for these baseline differences in subsequent analyses.

### Intervention effects on self-reported health outcomes

**Fig. 4:**
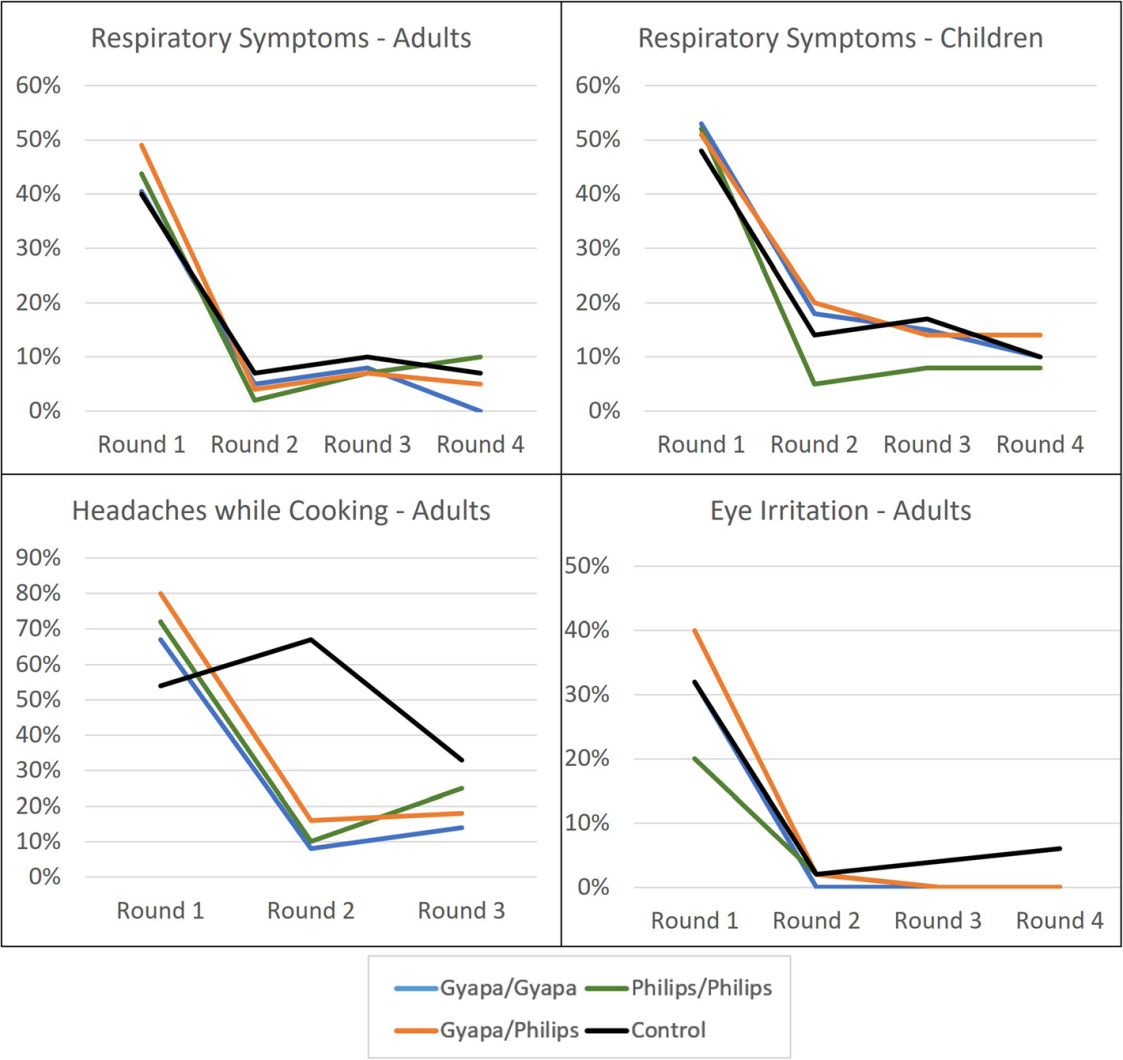
Self-reports of respiratory symptoms among adults and children (top), and headaches while cooking and eye irritation among adults (bottom), across survey rounds

Figure 4 plots the frequencies of self-reported health symptoms across time. We examine reports of any respiratory symptom in the past week, among both adults and children (top plots), as well as whether the respondent (adult primary cook) reported experiencing frequent headaches while cooking or eye irritation in the past week. We see a dramatic decrease in the frequency of any reported respiratory symptoms across all groups, including the control group, after the baseline round. For example, while 40–50% of adults reported having respiratory symptoms at baseline, this proportion is less than 10% across groups in all subsequent rounds. Chi-squared tests indicate no significant differences across groups in these reported frequencies in any survey round.

We also observe large decreases in reported frequencies of headaches and eye irritation among adults. For headaches, there is some evidence for improvements in the intervention groups relative to the control group, particularly in the second follow up round: 67% of control group cooks report experiencing headaches in this round, compared to 8% of Gyapa/Gyapa cooks, 11% of Philips/Philips cooks, and 15% of Gyapa/Philips cooks (*p* < 0.000). In the third follow up round, this difference attenuates substantially, due to both an increase in reported headache frequencies in the treatment groups, and a decrease in the control group, and the differences are no longer significant at the 10% level. (The headache question was inadvertently omitted from the fourth follow up survey). For eye irritation, the main observation is another large decrease in reported frequencies across all intervention groups. In particular, we observe that no cooks in the three intervention groups report eye irritation in the third and fourth follow ups, compared to 4–6% of control group cooks. As we discuss in more detail in the discussion section, we speculate that these large decreases in reported symptom frequencies may be due to a survey fatigue effect in which respondents avoided reporting symptoms to avoid lengthy follow-up questions.

### Intervention effects on biomarkers

**Fig. 5:**
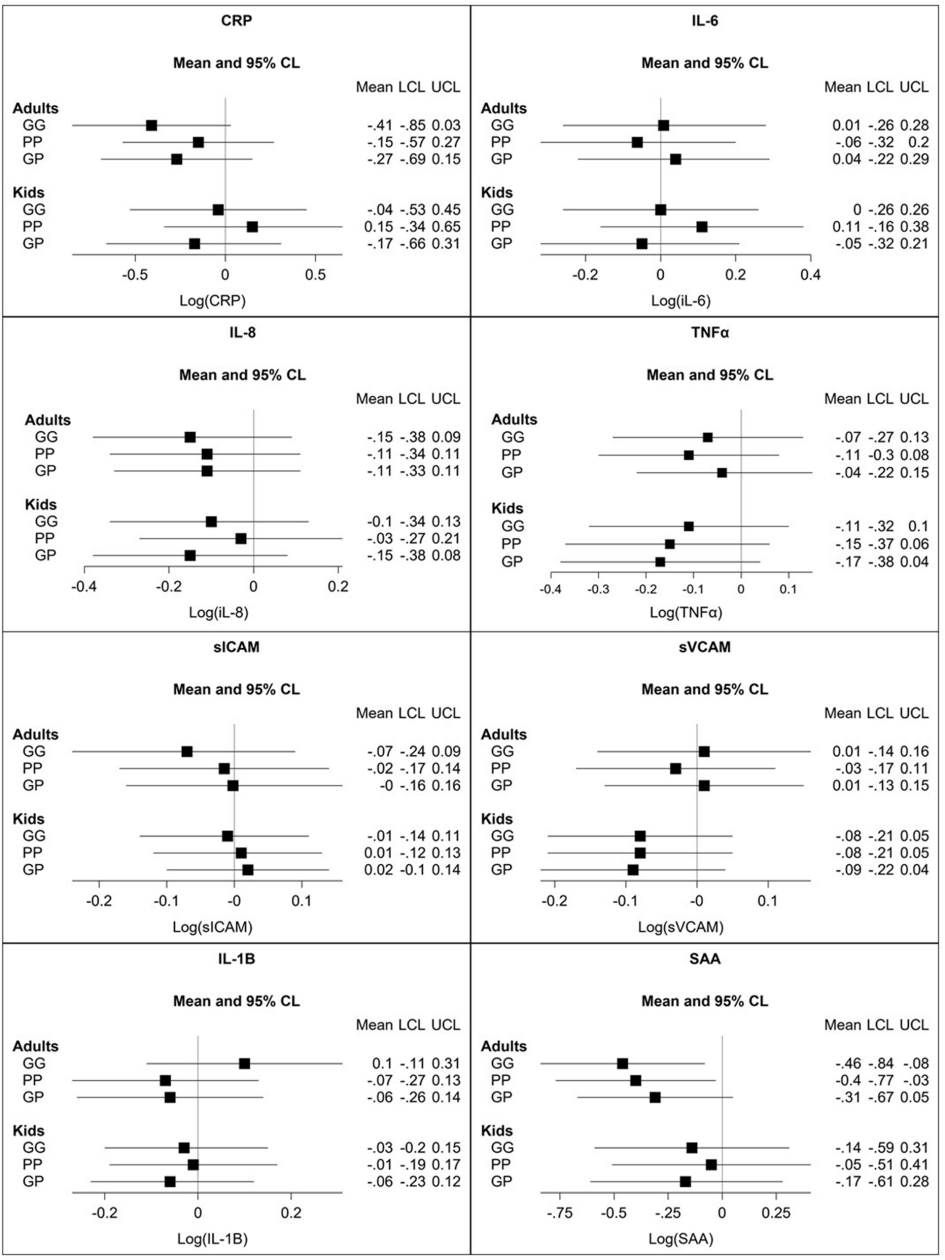
Coefficient estimates and 95% confidence intervals from linear mixed models examining the effect of the three stove interventions group on 8 biomarkers, relative to the control group. Each panel shows a separate biomarker, with results shown separately for adults (primary cooks, *n* = 184) and children (*n* = 211). Group GG = Gyapa/Gyapa, Group PP = Philips/Philips, Group GP = Gyapa/Philips (reference = Control). Covariates included are as shown in Eq. 1. Unadjusted results shown in Supplemental Fig. 1

**Fig. 6:**
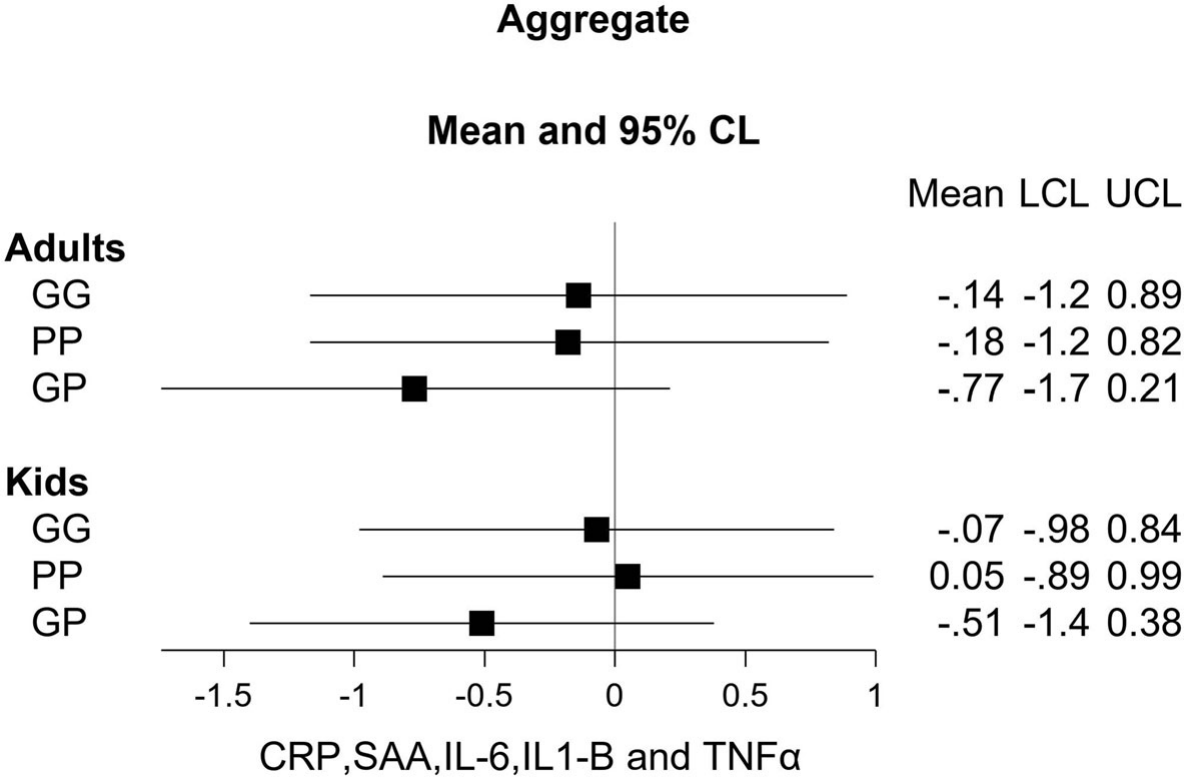
Coefficient estimates and 95% confidence intervals from linear mixed models examining the effect of the three stove interventions group on the aggregate biomarker score, relative to the control group. (Adults *n* = 184, kids *n* = 211). Group GG = Gyapa/Gyapa, Group PP = Philips/Philips, Group GP = Gyapa/Philips (reference = control). Covariates included are as shown in Eq. 1

Biomarker analyses were conducted for all individuals with measurements at baseline and in at least one of the three follow up rounds. Results from the linear mixed models for the 8 individual biomarkers for adults and children are shown in Fig. 5, and results for the aggregate biomarker outcome variable are shown in Fig. 6. Most of the point estimates are negative, indicating that inflammation levels were lower in the intervention groups compared to the control group. Results that meet or approach typical statistical significance levels include:

- CRP levels for adults: Gyapa/Gyapa group 0.41 ng/ml lower (*p* = 0.066) than control after adjusting for age and baseline BMI, household tobacco smoking, baseline CRP and season (log scale);
- SAA levels for adults: Gyapa/Gyapa 0.46 lower (*p* = 0.018), Philips/Philips 0.40 lower than control (*p* = 0.032), Gyapa/Philips 0.31 lower (*p* = 0.094) than control adjusting for age and baseline BMI, household tobacco smoking, baseline SAA and season (log scale);

There were no significant differences in the levels of the other biomarkers with regards to stove groups in either the adults or children. For the aggregate model, point estimates are again negative in most cases, but not statistically significant at conventional levels (Fig. 5). Using the Bonferroni-adjusted *p*-values (significance level of 0.002 for 24 comparisons), none of the biomarker results are statistically significant.

### Intervention effects on child growth

Child growth analyses were conducted on the sample of children who had anthropometric measurements collected at both baseline and endline (184 children in total, or 82.1% of children measured at baseline).

Although the overall models for the impact of stove group on each z-score were significant, the term for stove group itself was only significant for HAZ (Table 2). Philips/Philips had a higher endline HAZ than the Control group, while Philips/Gyapa had a lower endline HAZ than the Control Group. There was also no detectable influence of stove group on whether individuals were stunting (*p* = .23) or underweight (*p* = .56) at endline, with similar changes in proportion across all four groups. Although the overall underweight model was not significant, sex did emerge as a significant predictor with females being more likely to be underweight (*p* = .035). However, this is likely due to females generally having a lower birth weight than males [64, 65].

**Table 2:** Generalized Linear Models of stove group impact on anthropometric endline z-scores controlling for baseline, age and sex of individual

Response	Likelihood ratio chi-square	P-value	Significant Terms
HAZ	46.52	< .0001	Intercept, Baseline HAZ, Age, Stove Group B and C
WAZ	40.24	< .0001	Intercept, Baseline WAZ
MUACZ	31.21	< .0001	Baseline MUACZ

## Discussion

We find limited evidence that the REACCTING intervention improved targeted health outcomes among study participants. To begin, we examined a set of self-reported health symptoms over time across intervention groups. For respiratory symptoms, we found no evidence for differences across groups among adults or children. We observed large decreases in the frequency of these symptoms across *all* intervention groups over time, possibly due to a reporting or “survey fatigue” effect. Respondents that reported any symptoms were asked follow-up questions about treatment seeking; if respondents (or interviewers) sought to avoid these questions, they may have been less likely to report symptoms in later rounds. Given that data were collected over a two-year period and decreased in all follow up rounds, we do not believe these differences are due to seasonal effects. Overall, given apparent data quality issues, we do not place much weight on these self-reported health results.

Next, we examined a set of biomarkers that have been implicated in the inflammatory pathway linking exposure to PM with health outcomes. The most intriguing evidence for possible health effects involves adults’ CRP levels (lower in the Gyapa/Gyapa group relative to control) and SAA levels (lower in all three intervention groups relative to control). CRP and SAA have been linked to respiratory diseases including COPD [66], and CRP has also been associated with air pollution exposure and cardiovascular disease [19, 38]. A reduction in these inflammatory biomarkers and a trend in several other indicators provides suggestive evidence that intervention may have lowered the systemic pro-inflammatory status of these women. At the same time, in light of the number of tests that were run and the issue of multiple comparisons, we are cautious not to overinterpret our results. Any improvements that were achieved at the level of biomarkers did not manifest into improvements in child growth or self-reported health symptoms. Clinical outcomes (e.g., respiratory illness, mortality) were not measured in this study, and we would be underpowered to detect effects on these outcomes given our sample size.

Turning to child growth, although two stove groups significantly differed from the Control group in the endline HAZ model, this is likely an artifact of the sample. If stove group had a significant and realized impact, the parameter estimates for each stove group relative to the Control group should all be positive. Over time, individual HAZ tends to increase, especially mean HAZ for groups, sometimes irrespective of height deficits relative to a reference median increasing [67]. Since this is a low-income population, WHO Growth standards are somewhat biased as they were computed based on primarily Western populations, though no other appropriate references exist for this population at present.

It is informative to interpret these results in light of the study’s prior findings for measures along the causal pathway linking improved stove introduction to health outcomes. First, using both surveys and electronic stove use monitors, we found high levels of “stove stacking” (continued use of traditional stoves) across study groups throughout the study [7, 8]. Use of the lower-tech Gyapa stove was substantially higher than use of the more advanced Philips stove, and high rates of battery failure in the Philips stove contributed to a declining trend in use of this stove over time. Next, the health impacts of “improved” stoves are contingent on the extent to which these stoves reduce emissions relative to traditional stoves when operated by end-users in the field. The Philips stove in particular had shown promising emissions results in lab testing [52], which was a major factor in our decision to include this stove in our study (and in others’ decisions to use this stove in studies initiated around the same time [21, 68]). However, results from uncontrolled cooking tests (encompassing variability of a real world setting) showed mixed results across performance metrics [9]. As tested in the field, particulate matter emissions were not significantly reduced relative to a traditional three stone fire for either the Philips or the Gyapa stove, while the Philips stove did reduce carbon monoxide (CO) emissions by roughly 50% [9]. Finally, a growing body of evidence suggests that improved health outcomes are likely only realized with significant and substantial reductions in exposure to air pollution given a steep exposure-response curve [2, 69]. Personal exposure measures from the REACCTING study did show some reductions (49–63%) in organic carbon PM_2.5_ exposure for all intervention groups relative to the control, while elemental carbon PM_2.5_ exposure was reduced in groups receiving the Philips stove (44–59%) [11]. However, factors specifically linked to cooking / biomass burning (polycyclic aromatic hydrocarbons (PAHs) and methoxyphenol) did not differ significantly across intervention arms [11]. Additionally, source apportionment results from speciated personal and ambient PM_2.5_ point to large contributions of PM_2.5_ from sources other than stoves, reducing the potential for exposure reductions from a purely cookstove-targeted intervention [11]. Putting these pieces together, the story that emerges is that these stoves resulted in small reductions in exposure to HAP at best, leading to limited changes in the health measures examined here.

Our results are largely in line with the accumulating evidence from a broader collection of “improved” biomass stove studies [21, 23, 24, 70, 71] suggesting that low use levels, modest emissions and exposure reductions from “improved” stoves, and contributions of other community-level sources limit the potential for these interventions to substantially reduce the health burden related to HAP. There are exceptions to this general trend, including the RESPIRE study mentioned in the introduction [20]. A key difference between our study and RESPIRE is that the latter used a chimney stove in Guatemala, which was not feasible given kitchen design and cooking patterns in Ghana (e.g., both indoor and outdoor cooking). Closer to our study context, the Ghana Randomized Air Pollution and Health Study (GRAPHS) examined impacts on CO exposure and systolic blood pressure among pregnant women following an intervention that distributed improved biomass stoves (arm 1) and liquefied petroleum gas (LPG) (arm 2) stoves to participants [72]. Reductions in CO exposure were observed in the LPG arm only, and blood pressure was lower but not to a statistically significant degree across both study arms. Birthweight was not significantly different across study arms [3]. These results highlight that study context, cooking patterns, and the specifics of the cookstove intervention are important determinants of program impacts, and also that multiple potential health pathways (e.g., impacts on respiratory and cardiovascular health) should be considered.

The REACCTING study has several key limitations. First, the study was underpowered to assess impacts on clinical health endpoints and did not include these measures, limiting our ability to definitively determine whether or not these stoves improved participants’ health. As discussed above, the self-reported health measures we included did not generate reliable data on respondents’ health outcomes, and both models of “improved” cookstoves we employed ultimately showed limited real-world improvements over traditional stove models. We are also careful to note that these study’s findings may not generalize to all cookstove interventions or settings. The study population was randomly selected from the rural, biomass-reliant households of Ghana’s Kassena-Nankana Districts, such that results may generalize to this specific population and other similar populations in this region. However, a wide variety of factors, from the types of fuels and cookstoves used to cooking tasks performed, will influence the outcomes of cooking interventions in different settings.

## Conclusions

Recent enthusiasm for improved biomass cookstoves has been driven by their potential to provide win-win or triple-win scenarios, offering benefits for local users (health, time savings, development), local environments (reduced deforestation, local ambient air quality), and global climate [73, 74]. A growing number of studies, including this one, suggest that the human health impacts of these stoves should not be assumed. In light of this evidence, the health community and organizations like the Clean Cooking Alliance have shifted their focus to promotion of clean fuels like liquefied petroleum gas and electricity [75, 76], as well as advocating for community-level interventions to address both household-level and ambient air pollution sources [71].

Despite its limitations, we believe the REACCTING project provides a useful model for interdisciplinary, policy-relevant research in the household air pollution and household energy sectors. Households’ reliance on solid fuels to meet their basic energy needs continues to impose a significant health burden on populations around the world, along with environmental and social impacts. The evidence base to which this study contributes shows that simple solutions to this problem are elusive. Understanding and learning from both successes and failures requires expertise in a wide range of disciplines implicated in the causal pathway linking new technologies with desired impacts. Making progress towards transitions to cleaner household energy systems will require teams of social scientists, exposure scientists, environmental scientists, and health scientists working together, alongside affected community partners.

## Supplementary Information

**Additional file 1.**

**Additional file 2.**

## Data Availability

Available upon request from corresponding author.
