# Supplementary material for: Health impacts of a randomized biomass cookstove intervention in northern Ghana
Source: BMC Public Health. 2021 Dec 4;21:2211. doi: 10.1186/s12889-021-12164-y (PMC8642932; doi:10.1186/s12889-021-12164-y)
Supplement: Supplementary file 1 — Additional file 1. [file 12889_2021_12164_MOESM1_ESM.docx]

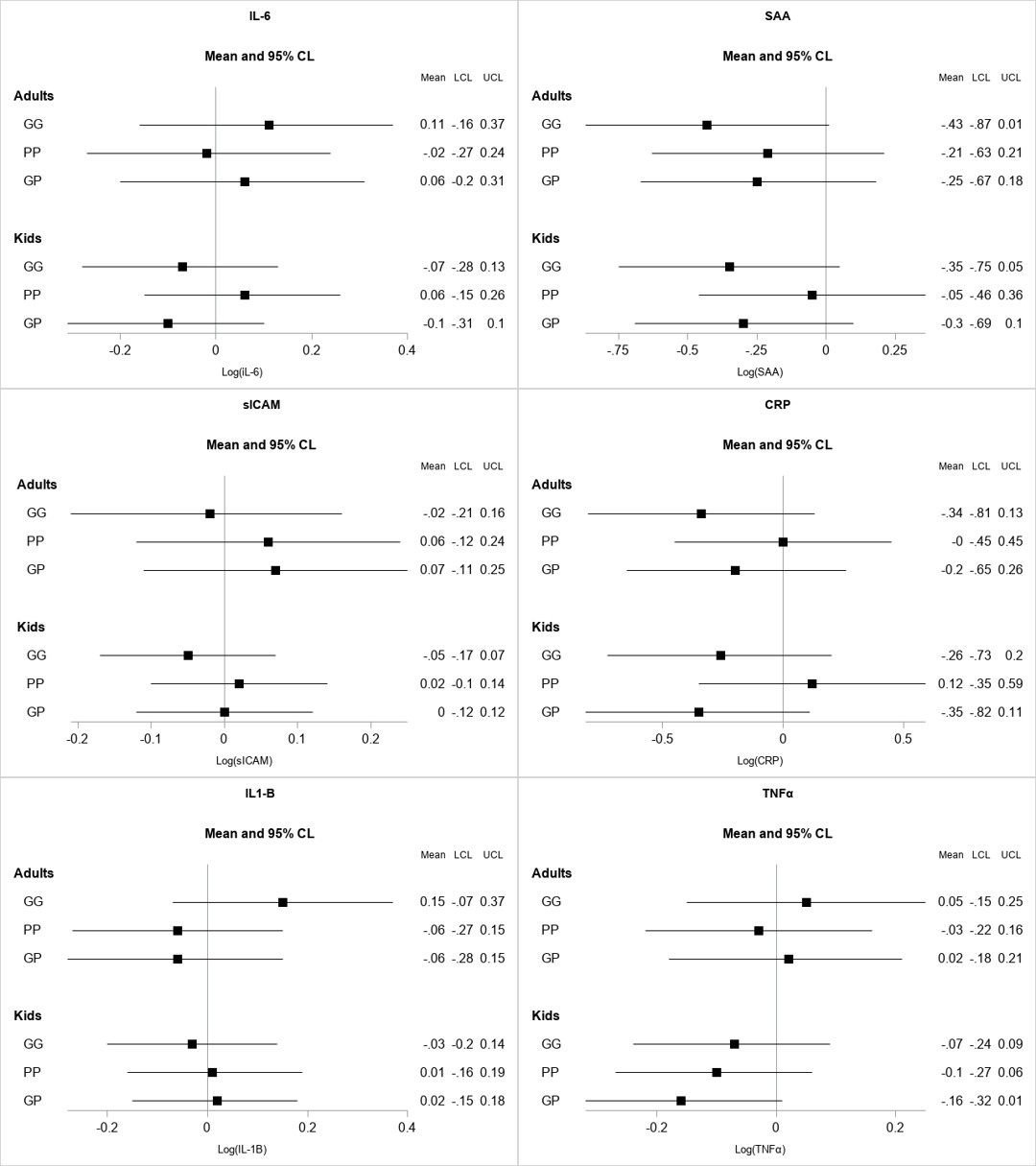


Supplemental Figure 1: Unadjusted coefficient estimates and 95% confidence intervals from linear mixed models examining the effect of the three stove interventions group on 8 biomarkers, relative to the control group. Each panel shows a separate biomarker, with results shown separately for adults (primary cooks, n=184) and children (n=211). Group GG = Gyapa/Gyapa, Group PP = Philips/Philips, Group GP = Gyapa/Philips (reference = Control). No additional covariates included.
